# Simultaneous development of adenocarcinoma and gastrointestinal stromal tumor (GIST) in the stomach: case report

**DOI:** 10.1186/1477-7819-10-6

**Published:** 2012-01-09

**Authors:** Daigo Yamamoto, Yoshinori Hamada, Yu Tsubota, Katsuhiro Kawakami, Chizuko Yamamoto, Mitsuo Yamamoto

**Affiliations:** 1 Department of Surgery, Kansai Medical University, Hirakata, Japan; 2 Department of Surgery, Seiko hospital, Neyagawa, Osaka, Japan; 3 Department of Neurosurgery, Seiko hospital, Neyagawa, Osaka, Japan; 4 Department of Internal Medicine, Seiko hospital, Neyagawa, Osaka, Japan

**Keywords:** gastric cancer, GIST, stomach

## Abstract

**Background:**

Gastrointestinal stromal tumors (GISTs) and adenocarcinoma are distinct neoplasms originating from different cell layers. Approximately 20% of patients with GIST develop other cancers.

**Case presentation:**

We report a case of the coexistence of adenocarcinoma and gastrointestinal stromal tumor (GIST). Gastric endoscopy showed the ulcerated tumor with bleeding along the lesser curvature of the proximal stomach and a submucosal nodule that measured about 3 cm in diameter in the lower part of the stomach body. Their pathological examination showed gastric cancer (poorly differentiated diffuse adenocarcinoma) and GIST (low-risk category). Further, immunohistochemical staining for C-kit and CD34 was positive, while that for SMA and S-100 was negative.

**Conclusion:**

Although it is not easy to speculate on the coexistence of adenocarcinoma and GIST, pre-and post-operative diagnoses may be essential, and such cancer development is not considered to be unusual.

## Background

In recent years, the synchronous occurrence of tumors of different histotypes arising in the same organ has been reported more frequently. Gastrointestinal stromal tumors (GISTs) and adenocarcinoma are distinct neoplasms originating from different cell layers. Although adenocarcinoma constitutes the most common type of gastric tumor, the synchronous development of a GIST is relatively rare [1-6]. Here, we present and discuss a case of synchronous gastric cancer and GIST.

## Case presentation

The patient was a 67-year-old man, who had been admitted to the hospital due to melena and a hematocrit level of 20.1 %. Nasogastric intubation revealed a fresh blood clot in the stomach. Urinalysis, chest and abdominal films, and liver function tests were within normal limits, and serum creatinine was 1.2 mg/dl. Five units of packed blood cells were transfused promptly.

**Figure 1:**
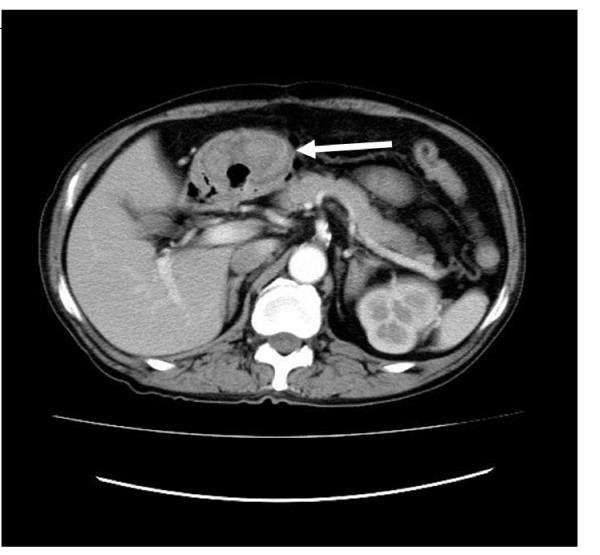
**An abdominal computed tomography (CT) scan showing a heterogeneous mass (3 cm × 4 cm) located in the lower part of the stomach body**. The tumor is indicated with an arrow.

**Figure 2:**
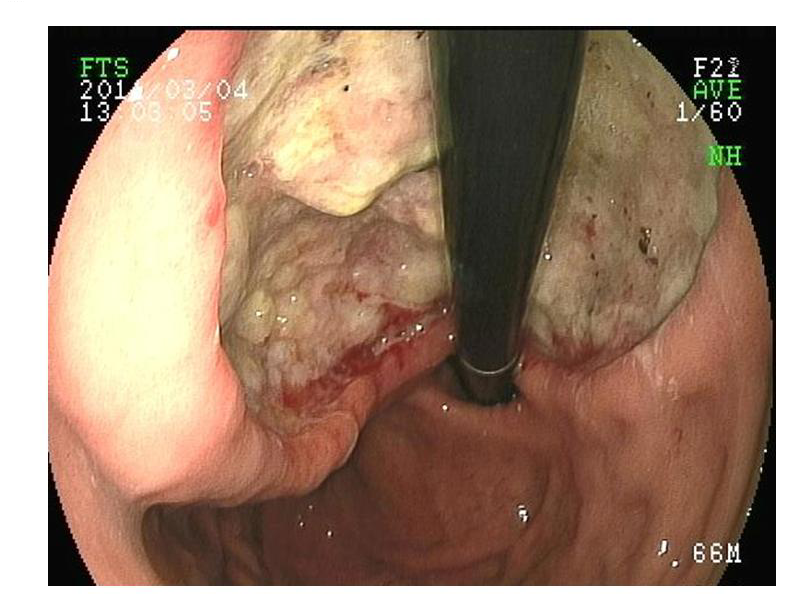
**Gastric endoscopy confirmed an ulcerated tumor with bleeding along the lesser curvature of the proximal stomach**.

**Figure 3:**
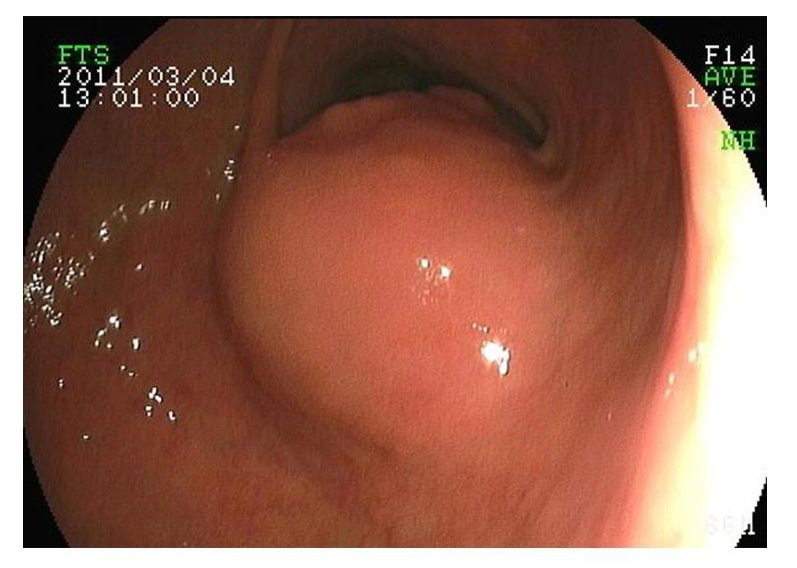
**Gastric endoscopy showing a submucosal nodule that measured about 3 cm in diameter in the lower part of the stomach body**.

**Figure 4:**
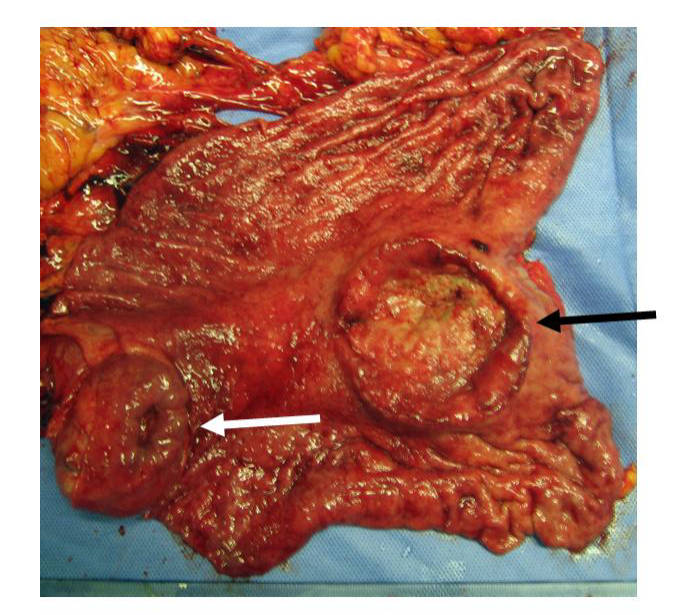
**Macroscopic examination of a total gastrectomy specimen showing Borrman type-2 tumor measuring 5 × 6 cm (black arrow) and submucosal nodule measuring 3 × 4 cm in the stomach (white arrow)**.

**Figure 5:**
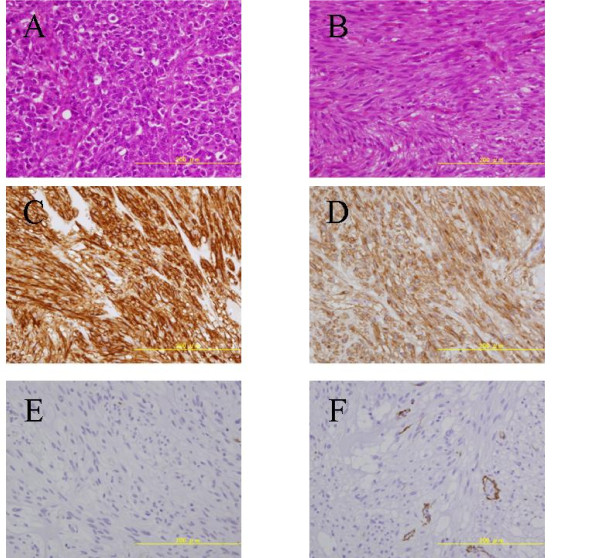
**Microscopic examination of the proximal tumor in the stomach demonstrating typical histological findings of adenocarcinoma (A)**. In addition, histopathological examination of the submucosal nodule revealed GIST of the low-risk category, composed of cytologically bland spindle cells with a low mitotic index (< 5) (B). Immunohistochemistry indicated strong staining for CD34 (C) and C-kit (D), while negative results were observed for S-100 (E) and SMA (F).

Abdominal CT scan showed a well-defined, heterogeneous mass (3 × 4 m) which was located in the lower part of the stomach body (Figure 1). Gastric endoscopy showed the ulcerated tumor with bleeding along the lesser curvature of the proximal stomach (Figure 2) and a submucosal nodule that measured about 3 cm in diameter in the lower part of the stomach body (Figure 3). No other metastatic lesions in other organs were found on abdominal ultrasonography or the CT scan. High-grade gastrointestinal bleeding persisted, necessitating the additional transfusion of ten units of packed red blood cells. Subsequently, the patient underwent a total gastrectomy. During the operation, there was no other evidence of metastatic disease in the intra-abdominal cavity. Macroscopic examination of the total gastrectomy specimen showed Borrman type-2 tumor measuring 5 × 6 cm and submucosal nodule measuring 3 × 4 cm in the stomach (Figure 4). On histopathological examination, the Borrman type-2 tumor gastric tumor showed transmural infiltration by a poorly differentiated diffuse adenocarcinoma (Figure 5A). There was no vascular invasion and no lymph node metastasis. Further histopathological examination of the submucosal nodule revealed GIST of the low-risk category [14], which was composed of cytologically bland spindle cells and showed a low mitotic index (< 5/50HPF).

The immunohistochemistry indicated strong staining for CD34 and C-kit, while expressions of SMA and S-100 were negative (Figure 5B-F). These findings confirmed the simultaneous development of gastric cancer and GIST. The patient was subsequently discharged without any complications.

## Discussion

The coexistence of adenocarcinoma and GIST is uncommon, and GIST has often been detected incidentally in the gastric mucosa or serosa, or, occasionally, intramurally, at surgery [1-4]. Further, gastric tumors are derived from various other carcinomas and a precise pre- and post-operative diagnosis is important. However, when the GIST is submucosal or subserosal, the gastric mucosa may not be invaded, and endoscopic biopsies can be normal. In fact, in our case, the pre-operative biopsy fragments showed only adenocarcinoma, and the GIST was detected only in the resected stomach. Therefore, it may be difficult to diagnose preoperatively.

Approximately 20% of patients with GIST develop other cancers and various hypotheses have been proposed regarding the simultaneous development of GIST and adenocarcinoma [7-9]. It is not clear whether this is a simple incidental coexistence or the two lesions are connected by a causal relationship. Gene mutations may underlie tumor predisposition in patients harboring a double neoplasia. However, at present, no data are available to support such a hypothesis.

GIST is the most common mesenchymal tumor [10], accounting for about 0.1 to 3% of all GI tumors [11]. IHC staining, such as is for CD34, smooth muscle actin (SMA), and S100, as well as c-kit (CD117), is necessary to make an accurate diagnosis of GIST [13]. It was revealed that c-kit and CD34 showed diffuse, strongly positive expressions in GIST. Rabin et al. [11] reported that 40 to 70% of GIST's were positive for CD34, 20 to 30% were positive for SMA, and 10% were positive for S100 protein [12,13]. Therefore, immunostaining of CD34, c-kit, SMA, and S100 is useful, and we could confirm the histological diagnosis using these markers. Accordingly, immunohistochemical as well as clinical information may be required in order to diagnose GIST appropriately. Although it is not easy to speculate on the coexistence of adenocarcinoma and GIST, pre- and post-operative diagnoses may be essential. Further the adjuvant therapy and lymphadenectomy are important. The refinement of risk stratification systems [14] will increase the precision of these systems for predicting recurrence, which may facilitate improvements in individual disease management.

## Consent

Written consent was obtained from the patient for publication of this study and the related photos.

## Competing interests

The authors declare that they have no competing interests.

## Authors' contributions

The authors(s) wrote the original manuscript.
